# Demand for emergency services during the COVID-19 pandemic and disease burden: a case study in Portugal

**DOI:** 10.3389/fpubh.2023.1294204

**Published:** 2024-01-15

**Authors:** Alcina Nunes, Catarina Costa, João P. Martins, Pedro L. Ferreira, Rui Pimenta

**Affiliations:** ^1^UNIAG, Instituto Politécnico de Bragança, Bragança, Portugal; ^2^Centro Hospitalar Universitário do Porto, EPE, Porto, Portugal; ^3^Escola Superior de Saúde, Instituto Politécnico do Porto, Rua Dr. António Bernardino de Almeida, 4200-072 Porto, Portugal; ^4^CEAUL – Centro de Estatística e Aplicações, Faculdade de Ciências, Universidade de Lisboa, Lisboa, Portugal; ^5^Faculty of Economics, University of Coimbra, Coimbra, Portugal; ^6^Centre for Health Studies and Research of University of Coimbra, Centre for Innovative Biomedicine and Biotechnology, Coimbra, Portugal

**Keywords:** emergency service, pandemic (COVID-19), user profile, disease burden, panel (longitudinal) data analysis

## Abstract

**Background:**

The COVID-19 pandemic brought changes in the pattern of care use. A significant increase in the volume of emergencies was expected. However, a significant decrease was observed worldwide.

**Methods:**

An observational, analytical and cross-sectional study of all records of emergency episodes of patients aged 18 years or older admitted to the emergency services of the University of Porto Hospital Centre (2018–2022) were analysed.

**Results:**

During the pandemic, a significant reduction in emergency episode admissions (up to 40% during lockdowns), an increase in pre-emergency services, and discharges from Infectious Diseases and Internal Medicine was observed. The discharges from General Practice and General Practice and Family Medicine were residual.

**Conclusion:**

The lower use and type of use of emergency services during the COVID-19 pandemic had a negative impact on the disease burden. This could be prevented in future pandemics through the development of strategies to promote confidence in the use of health resources and establishing contingency plans for virtual assistance.

## Background

The recent COVID-19 pandemic has led to changes in the pattern of health care use around the world. As emergency services (ES) are a central element in immediate health care, they are sensitive to external changes caused by epidemiological events, such as the (extreme) case of a pandemic. These events create an increase demand pressure that would cause an even greater overload on these services. In these critical response periods, emergency services’ typical characteristics also change, with even greater prioritisation of episodes with greater severity and mortality, which may result in less attention to other diseases, leading to a more significant disease burden.

Portugal has the highest rate of use of ES *per capita* among the Organization for Economic Cooperation and Development (OECD) countries, more than 70 visits per 100 inhabitants, which is significantly higher than the OECD mean value of 31 per 100 inhabitants (1). It is expected, at first sight, that a pandemic situation increases the amount of emergency episodes, since the health services would have to attend to an additional volume of cases associated with the new disease condition. However, contrary to this perception, studies on the impact of the pandemic on the use of health services have shown a very significant decrease in the volume of emergency care. This trend was consistent across countries (2–6). In Portugal, according to studies carried out in the first month of emergency state, this trend towards a decrease in emergency episodes was also recorded (7, 8).

Determining the optimal level of utilisation of health services is a complex task. The recent pandemic has brought changes in the behaviour of users of emergency services. However, it is important to assess whether the reduction in urgency episodes could be a problem in the short/medium term with the increase in the stock of disease that could lead to later, more severe urgency episodes.

The moral conscience of not overloading services focused on responding to the pandemic, travel restrictions and social isolation/quarantine, difficulties in finding transportation, or even avoiding the risk of contamination may justify the option of not consuming urgency/emergency resources.

This work analyses the access to the ES of the University of Porto Hospital Centre (CHUPorto) located in the North of Portugal. This highly differentiated unit was in the first line of response when it comes to the emergence of new infections, as there are no clear protocols, at least in the first phase, to deal with a situation that arises for the first time. Thus, the recent COVID-19 pandemic situation required CHUPorto to put in place a series of measures and restructuring, following government recommendations (9). The non-urgent care activity of outpatient consultation, scheduled and outpatient hospitalisation, complementary means of diagnosis and therapy, and scheduled surgery, was suspended/cancelled as long as it did not compromise the clinical status of the patient. When the pandemic situation allowed it, the non-urgent/scheduled assistance activity was resumed following the necessary contagion prevention measures. In the ES, an independent circuit was created with a permanent team dedicated to suspected cases of SARS-COV-2 infection. An internment dedicated exclusively to COVID-19 patients was structured on an isolated floor, which was under the care of the Infectious Diseases service. Also in the Intensive Care Unit, an isolated area dedicated to COVID-19 patients in critical condition was established.

Hence, through the development of an observational, analytical, and cross-sectional study, this work aims to analyse: (i) The distribution of urgent and non-urgent episodes during the pre and during pandemic periods; (ii) The profile of ES users and characteristics of emergency episodes and assessment of their changes throughout the pandemic; and (iii) The impact that the restrictions imposed during lockdown/confinement periods had on access to the ES.

## Methods

### Data collection

A proposal for carrying out the present study was submitted to the competent authorities of the CHUPorto. Authorisation from the Board of Directors of CHUPorto was obtained on 24 Aug 2022. The database of the current study includes administrative information related to emergency episodes. All the information was dealt according to Regulation 2016/679 of the European Parliament and of the Council of 27 April 2016. This means that it was provided anonimously.

Data analysis was proceeded by a data cleansing where data that met at least one of the following exclusion criteria was eliminated: (i) Duplicated episode; (ii) Episode without age information; (iii) Episode related to children aged under 18 years (at the time of admission); and (iv) Sufficient Information not available about the episode triage according to the Manchester Triage System (MTS). Triages results as no colour or white colour were not used.

**Figure 1:**
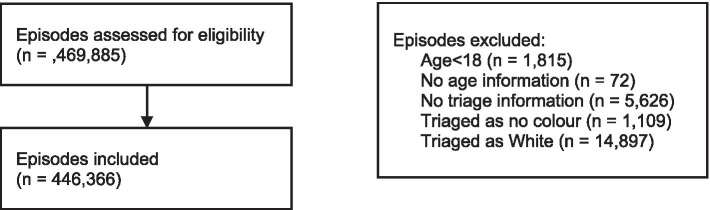
Flow chart showing the selection of episodes.

From an initial number of 469,885 emergency episodes, 23,519 were deleted. Thus, 446,366 episodes were considered for data analysis (further details are presented in Figure 1).

### Statistical analysis

Data were expressed as mean and standard deviation (SD) or as counts and percentages. Statistical analysis was performed using IBM SPSS Statistics^®^ 28 software.

Comparison of means was performed through a one-way ANOVA with Tukey’s *post hoc* multiple comparison tests. When the homogeneity of variances assumption did not hold, the Welch test was applied. Chi-square tests were applied to compare the percentages observed in each period. Statistical tests were performed at a significance level of 5%.

## Results

The 446,366 urgency episodes were recorded over four years (from March 10th 2018 to March 9th 2022). The last two years of this period were lived amid the COVID-19 pandemic. During this period, various restrictions were implemented to control the evolution of the pandemic (for example, mandatory use of a mask in specific contexts or restrictions on freedom of movement). These restrictions were especially intense during two lockdowns in which the country lived under a state of emergency and will be designated as periods of confinement. The 2nd period was much longer than the 1st period, which led us to divide this period into two periods of equal length. Thus, the four years under analysis were subdivided into five periods:

- Pre_P: pre-pandemic (722 days, from March 10th 2018 to February 29th 2020)
- C1: 1st confinement (46 days, from March 18th 2020 to May 2nd 2020)
- C2,1: first half of the 2nd confinement (55 days, from January 13th 2021 to March 8th 2021)
- C2,2: second half of the 2nd confinement (55 days, from March 9th 2021 to May 2nd 2021)
- P: pandemic period excluding the periods of confinement (583 days, the remaining dates)

Table 1 summarises some general characteristics of the individuals in these five periods. Three age groups were considered: young adults (18–30 years old), adults (31–65 years old) and older adults (over 65 years old).

**Table 1:** Characteristics of the included emergency episodes.

		Pre_P		C1		C2,1		C2,2		P	
Episodes	Mean SD	339.9_a_	46.2	161.1_b_	37.1	207.8_c_	36.3	263.4_d_	37.3	287.6_e_	51.5
Gender	Female	130260_a_	53.1	3651_b_	49.3	5688_b_	49.8	7617_a,c_	52.6	87259_c_	52.1
	Male	115133_a_	46.9	3761_b_	50.7	5743_b_	50.2	6,871 _a,c_	47.4	80383_c_	47.9
	Total	245,393		7,412		11,413		14,488		167,642	
COVID-19	Positive	0_a_	0	51_b_	0.7	708_c_	6.2	117_b_	0.8	4942_d_	1.3
	Negative	245393_a_	100	7361_b_	99.3	10723_c_	93.8	14371_b_	99.2	162700_d_	98.7
Age	Mean SD	55.5_a_	20.2	57.2_b_	19.5	57.5_b_	20.2	57.0_b_	20.0	55.t_a_	20.4
	18–30	37637_a_	15.3	851_b_	11.5	1485_c_	13.0	1938_c_	13.4	25345_a_	15.4
	31–65	118898_a_	48.5	3725_b_	50.3	5351_c_	46.8	6884_a,c_	47.5	78930_c_	47.9
	>65	88,858 _a_	36.2	2836_b,c_	38.3	4595_c_	40.2	5666_c_	39.1	60592_b_	36.8
Residence	Zone 1	144441_a_	61.2	4806_b_	65.7	7182_c_	63.5	9095_c_	63.4	102908_c_	63.0
	Zone 2	65699_a_	27.8	1831_b_	25.0	2960_b,c_	26.2	3772_b,c_	26.3	43454_c_	26.6
	Zone 3	13186_a_	5.6	299_b_	4.1	567_a,c_	5.0	717_c_	5.0	7852_c_	4.8
	Zone 4	12604_a_	5.3	378_a,b_	5.2	610_a,b_	5.4	758 _a,b_	5.3	9230_b_	5.6
Source	External	161472_a_	65.8	787_b_	51.1	6307_c_	55.2	8818_d_	60.9	101342_d_	60.5
	INEM	54273_a_	22.1	2216_b_	29.9	3267_b_	28.6	3542_c_	24.4	43539_d_	26.0
	PCS	12369_a_	5.0	359_a,b_	4.8	675_c_	5.9	789_a,c_	5.4	7647_b_	4.6
	Public hospital	8812_a_	3.6	237_a_	3.2	478_b_	4.2	522_a,b_	3.6	5909_a_	3.5
	SNS 24	6513_a_	2.7	738_b_	10.0	547_c_	4.8	703_c_	4.9	7692_c_	4.8
	Other	1954_a_	0.8	75_a,b,c_	1.0	157_c_	1.4	114_a,b_	0.8	1513_b_	0.9
STM wristband	Red	1585_a_	0.6	44_a,b_	0.6	77_a,b_	0.7	74_a,b_	0.5	949_b_	0.6
	Orange	30122_a_	12.3	1113_b_	15.0	1662_b_	14.5	1533_c_	10.6	18230_c_	10.9
	Yellow	166680_a_	67.9	4649_b_	62.7	6904_c_	60.4	8735_c_	60.3	101858_c_	60.8
	Green	42572_a_	17.3	1437_b_	19.4	2575_c_	22.5	3818_d_	26.4	42591_d_	25.4
	Blue	4434_a_	1.8	169_b,c_	2.3	213_a,c_	1.9	328_b,c_	2.3	4014_b_	2.4
Outcome	Discharge	129198_a_	52.7	2926_b_	39.5	4928_c_	43.1	6844_d_	47.2	77619_d_	46.3
	Sup. discharge	72281_a_	29.5	2795_b_	37.7	3934_c_	34.4	4988_c_	34.	58198_c_	34.7
	Hospitalisation	29981_a_	12.2	1432_b_	19.3	2250_b_	19.7	2126_c_	14.7	22546_d_	13.4
	Dropout	12095_a_	4.9	176_b,c_	2.4	208_c_	1.8	386_b_	2.7	7784_d_	4.6
	Walkout	1169_a_	0.5	35_a,b,c_	0.5	49_a,c_	0.4	101_b_	0.7	954_b,c_	0.6
	Death	615_a_	0.3	48_b_	0.6	62_b_	0.5	41_a,c_	0.3	538_c_	0.3
Week	Weekdays	184341_a_	75.1	5597_a_	75.5	8668_a_	75.8	10896_a_	75.2	126320_a_	75.4
	Weekend	61052_a_	24.9	1815_a_	24.5	2763_a_	24.2	3592_a_	24.8	41322_a_	24.6
Day	8 am to 8 pm	181922_a_	74.1	5417_a_	73.1	8791_b_	76.9	11064_b_	76.4	125163_c_	74.7
	8 pm to 8 am	63471_a_	25.9	1995_a_	26.9	2640_b_	23.1	3423_b_	23.6	42479_c_	25.3

Pairwise comparison is provided using subscript letters. If a pair has any subscript letter in common, then the difference is not significant at a 0.05 level. A combination of two or more subscript letters in a single category means that there are no significant differences with two or more other categories (however, these categories are significantly different from each other). Data are No. and percentage unless stated otherwise. The numbers do not always sum to group totals due to missing information for some variables. Pre_P: Pre-pandemic. C1: 1st confinement. C2,1: First half of the 2nd confinement. C2,2: Second half of the 2nd confinement. P: Pandemic period excluding the periods of confinement.

The places of residence were grouped into four zones. Every individual was placed in the first zone whose criteria were fulfilled:

- Zone 1: Resident in an area where the CHUPorto is a first-line hospital in terms of hospital response (municipality of Gondomar and municipality of Porto, except for the parishes of Bonfim, Campanhã and Paranhos);
- Zone 2: Resident in the PMA (Porto Metropolitan Area);
- Zone 3: Resident in an area where CHUPorto is the highly differentiated reference hospital (districts of Vila Real and Bragança, municipalities in the north of the district of Aveiro and Viseu and municipalities of Amarante, Baião and Marco de Canaveses);
- Zone 4: Residents in the remaining Portuguese territory.

The agent who determined the access to the ES is specific to a particular variable source. It can be a health institution or individuals (external). Health institutions comprehend a wide range of services: (i) SNS24 – a telephone triage service in Portugal that is part of the country’s National Health Service; (ii) INEM – the organisation responsible for coordinating and providing medical emergency services throughout the country; (iii) PCS – Primary Care Services, Private Health Institutions; (iv) other National Health Service hospital; or (v) a CHUPorto’s inpatient service.

The episode’s outcome was defined as: (i) regular discharge, (ii) supervised discharge, (iii) dropout, (iv) walkout (exit against medical opinion), (v) hospitalisation or (vi) death. The supervised discharge includes the recommendation to proceed with medical care at other health institutions.

The week and day variables make it possible to cross-reference episode admissions with the hours of operation of the CSP (usually Monday through Friday from 8 am to 8 pm).

The daily mean of emergency episodes before the pandemic was around 340. The surge of the pandemic (period C1) resulted in a drop to less than half (around 160). In the 2nd confinement, the number of occurrences presents a smother drop. However, in its first half, there is a reduction of almost 40% compared to the pre-pandemic period. In the other pandemic periods, the number of episodes reduction was around 15%.

**Figure 2:**
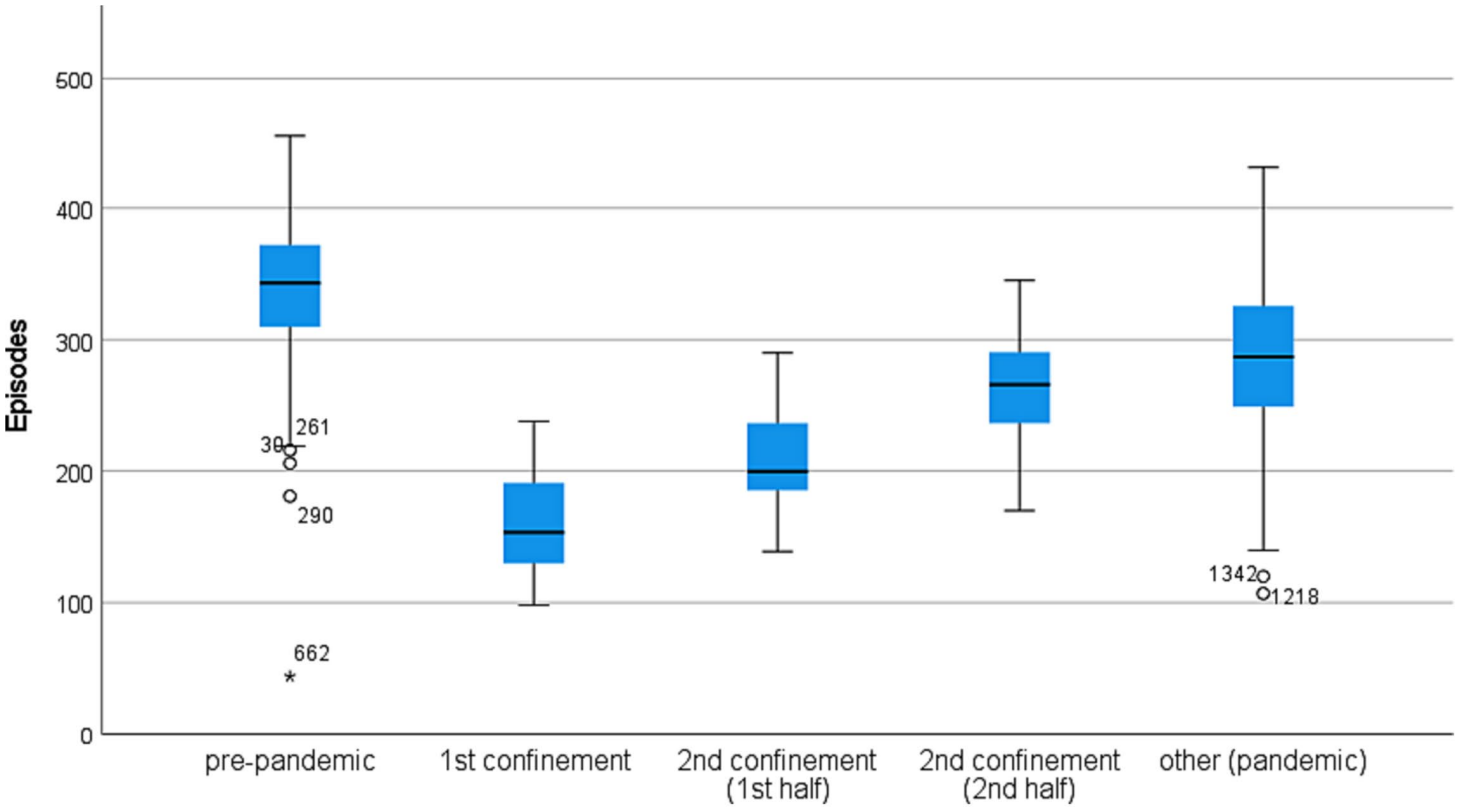
Daily episodes by each period. (The numbers associated with the outliers correspond to the number of days counted since the beginning of the study; all pairwise comparisons are significantly different at a 0.05 level).

This variation in the maximum daily number of episodes in the five periods considered was noticeable (see Figure 2). In the 1st confinement and the 1st half of the 2nd confinement, the maximum daily number of emergency episodes reached was even below the maximum value of 25% of days, with the lowest demand in the pre-pandemic period.

The greatest dispersion in demand is found in the pandemic period (excluding confinements). The minimum is observed in the pre-pandemic period (day 662), corresponding to the last day of 2019.

The number of episodes decreased during the pandemic, especially during the confinements. More than 75% of the days of the 1st confinement had a number of episodes below 200 (see Figure 2). The number of episodes was also below 200 in half of the days of the first part of the 2nd confinement. In the other pandemic periods, the values are closer to the pre-pandemic. The demand exceeded 200 episodes on more than 75% of those days.

Given that demand has undergone significant variations in quantitative terms, it is critical to analyse whether this variation also extends to the composition of ES demand.

The age of the individuals demanding for ES underwent a significant change during the periods of confinement, with the mean age increasing by up to a maximum of 2 years when compared to the pre-pandemic period. A considerable increase in the percentage of demand for ES by adults over 65 years old helps to explain that increase. On the other hand, the percentage of demand by young people (18–30 years) decreased significantly during the periods of confinement.

The drop in demand for ES during the pandemic also resulted in significant variations in individuals’ places of residence, especially during the 1st confinement. During this period, the percentage of users in Zone 1 increased significantly. This variation was accompanied by a significant reduction in demand from individuals living close to the hospital unit (Zone 2) as well as from reference areas for care with a high degree of differentiation (Zone 3). Only the percentage of individuals from other areas (Zone 4) did not change significantly. The 2nd confinement did not show significantly different values from the other pandemic periods (P).

Regarding the agent who determined access to the ES, the pandemic is characterised by some significant changes. One of the most notorious is the considerable drop in the demand for individuals who arrive at the emergency service by themselves (external) during the 1st confinement (the percentage decreases by about 15%). On the other hand, the number of individuals accessing through INEM and SNS 24 (telephone line) increased during the pandemic. In the latter case, the behaviour was quite disparate during the pandemic. If, during the 1st confinement, the percentage of patients indicated by the SNS 24 almost quadrupled, going from the 5th to the 3rd largest source of patients, the values for that period were reduced to about half in the remaining periods associated with the pandemic.

The episodes emergency level measured by the wristband colour attributed after the MTS – presented a different pattern during the pandemic. There was an increase in the percentage of green wristbands during all pandemic periods to the detriment of a decrease in the percentage of yellow wristbands. The variation in the percentage of orange wristbands was variable, with values above the pre-pandemic period during the 1st and first half of the 2nd confinement and below in the remaining periods of the pandemic.

**Figure 3:**
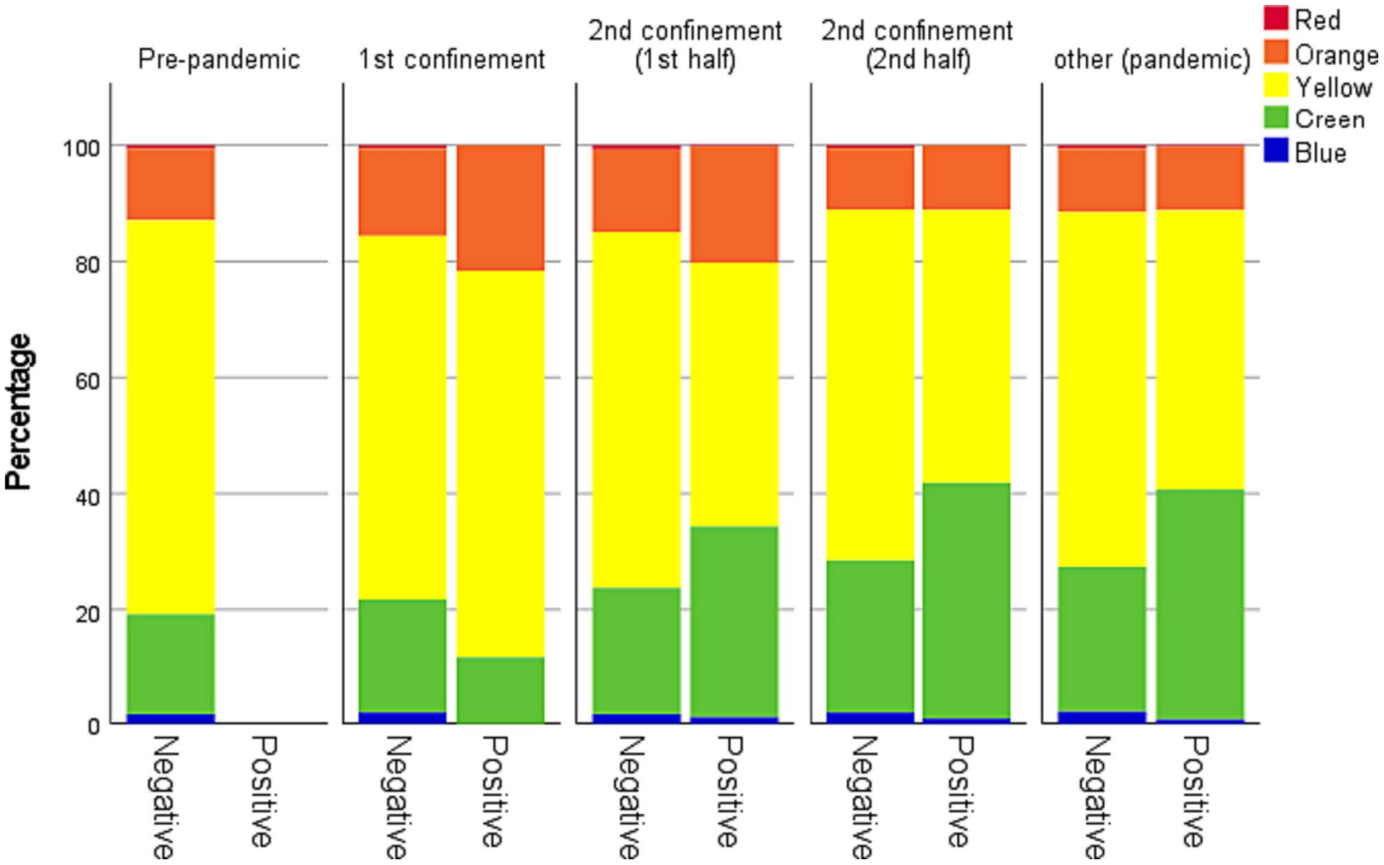
Percentage of STM wristbands by colour (COVID-19 positive versus COVID-19 negative individuals). All pairwise comparisons between the percentage of COVID-19 positive individuals are significantly different at a 0.05 level unless between the 1st confinement and the 2nd half of the 2nd confinement (*p* = 0.338).

Figure 3 compares this variation for patients diagnosed with COVID-19 and others. During the 1st confinement, the wristbands assigned to these two groups are very similar. In other periods, it appears that patients with COVID-19 have a level of emergency lower than other individuals (lower percentage of yellow wristbands).

The outcome of the individuals who resorted to the CHUPorto ES varied significantly with the onset of the pandemic. More than half of cases in the pre-pandemic period resulted in (regular) discharge. During the pandemic, this situation changed, with that percentage dropping to around 40% in the 1st and first half of the 2nd confinement. Moreover, in the 1st confinement, the discharges and supervised discharges were very close (difference less than 2%), while it was greater than 20% in the pre-pandemic period. The percentage of situations that resulted in hospitalisation also increased significantly during the pandemic, especially during (once again) the 1st and first half of the 2nd confinement, with hospitalisations increasing by more than 50% to around 20%. Concerning the episodes that ended with individual death, it is observed that during the 1st confinement, the percentage of fatalities doubled. This situation was not observed in the other periods. The dropout of the ES dropped more than 50% during the pandemic.

**Figure 4:**
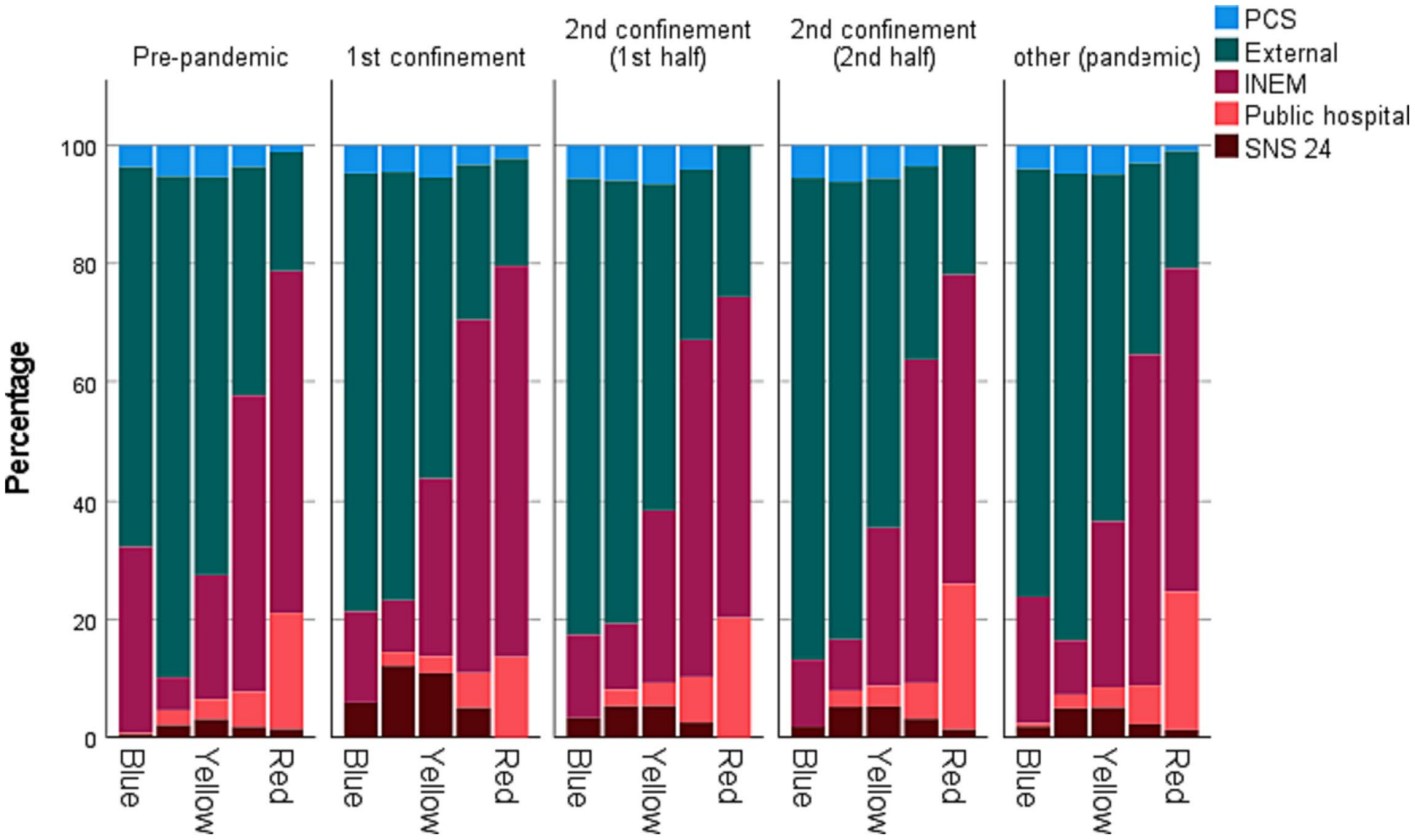
Percentage of STM wristbands according to the individual provenance.

In Figure 4, it is possible to verify that the decrease in individuals screened with a yellow wristband is justified by the decline in the demand of individuals who arrive at the ES by themselves. Although individuals who have come through INEM and SNS 24 have increased (in percentage) during the 1st confinement. Further details can be found in Table 1.

Table 2 describes the number of specialities responsible for discharge at CHUPorto during the period under review was 32. As 15 are each responsible for a maximum of 0.3% of discharges, they were grouped in the Other category.

**Table 2:** CHUPorto speciality responsible for hospital discharge.

		Pre_P		C1		C2,1		C2,2		P	
Speciality	Cardiology	1259_a_	0.5	36_a,b_	0.5	39_a,b_	0.3	46_b_	0.3	560_b_	0.3
	Dermatology	941_a_	0.4	1_b_	0.0	3_b_	0.0	10_b_	0.1	150_b_	0.1
	Family medicine	4201_a_	1.7	2_b_	0.0	1_b_	0.0	1_b_	0.0	27_b_	0.0
	Gastroenterology	1461_a_	0.6	42_a,b_	0.6	91_a,b_	0.8	118_b_	0.8	1096_a,b_	0.7
	General practice	40954_a_	16.7	735_b_	9.9	1170_b_	10.2	2038_c_	14.1	22950_c_	13.7
	General surgery	36339_a_	14.8	1090_a,b_	14.7	1599_a_	14.0	2205_a,b_	15.2	25826_b_	15.4
	Infectious diseases	944_a_	0.4	850_b_	11.5	858_c_	7.5	200_d_	1.4	5834_e_	3.5
	Internal medicine	55365_a_	22.6	2283_b_	30.8	3429_b,c_	30.0	4177_c_	28.8	49059_c_	29.3
	Nephrology	2035_a_	0.8	29_b_	0.4	57_b_	0.5	52_b_	0.4	848_b_	0.5
	Neurosurgery	3517_a_	1.4	116_a_	1.6	167_a_	1.5	199_a_	1.4	2273_a_	1.4
	Neurology	7521_a_	3.1	194_a,b_	2.6	331_a,b_	2.9	436_a,b_	3.0	4406_b_	2.6
	Otorhinolaryngology	14230_a_	5.8	285_b_	3.8	572_c_	5.0	798_a,c_	5.5	8912_c_	5.3
	Ophthalmology	24770_a_	10.1	634_b_	8.6	1027_b,c_	9.0	1435_a,c_	9.9	14766_b_	8.8
	Orthopedics	31198_a_	12.7	561_b_	7.6	1243_c_	10.9	1681_c_	11.6	19609_c_	11.7
	Stomatology	1764_a_	0.7	50_a,b_	0.7	72_a,b_	0.6	114_a,b_	0.8	1465_b_	0.9
	Urology	8422_a_	3.4	274_a_	3.7	388_a_	3.4	514_a_	3.5	5531_a_	3.3
	Vascular surgery	5122_a_	2.1	168_a,b,c_	2.3	295_c_	2.6	364_c_	2.5	3143_b_	1.9
	Other	2986_a_	1.2	58_b_	0.8	82_b_	0.7	96_b_	0.7	1119_b_	0.7
	Missing	2364_a_	1.0	4_b_	0.1	7_b_	0.1	4_b_	0.0	68_b_	0.0
Total		245,393	100	7,412	100	11,431	100	14,488	100	167,642	100

Pairwise comparison is provided using subscript letters. If a pair has any subscript letter in common, then the difference is not significant at a 0.05 level. A combination of two or more subscript letters in a single category means that there are no significant differences with two or more other categories (however, these categories are significantly different from each other). Data are No. and percentage unless stated otherwise. The numbers do not always sum to group totals due to missing information for some variables. Pre_P: Pre-pandemic. C1: 1st confinement. C2,1: First half of the 2nd confinement. C2,2: Second half of the 2nd confinement. P: Pandemic period excluding the periods of confinement.

In fact, the percentage of discharge attributed to each speciality varied significantly in some cases. The specialities of Infectious Diseases and Internal Medicine associated with treating patients with COVID-19 have increased considerably. In the case of Internal Medicine, just over 20% of discharges were attributed to this speciality in the pre-pandemic period. During the pandemic, this figure has always been around 30%. In the case of Infectious Diseases, in the pre-pandemic period, discharges from this speciality were almost residual (0.4%). In the 1st confinement, that percentage increased to nearly 30 times more!

Despite the increase in discharges attributed to these specialities, there was no uniform decrease in the other specialities. Some did not even have significant variations in percentage terms. General Practice, Dermatology, Family Medicine, and Nephrology specialities present higher drops. The cases of Family Medicine and Dermatology are relevant as demand during confinement periods was practically null.

The percentage of demand during the week and weekends did not vary significantly in the considered periods. Regarding access times, there is a significant decrease in demand at night during the 2nd confinement.

## Discussion

The pandemic led to changes in the pattern of use of health resources, and worldwide there was a downward trend in the volume of emergencies. In our study, the same tendency was registered. A significant reduction in hospitalisations was observed, especially during the 1st confinement, with a drop of more than 50%. This pattern can be explained by the “fear” of the new disease. On the 1st day of C1, only one death was recorded in Portugal due to the COVID-19 disease and the number of new cases on that day was less than 200 (regardless of the severity of the cases). Pressure on ES was never high and the lockdown measures took place before the epidemic curve grew (10). Thus, a significant decrease in the percentage of yellow bracelets is observed, as lockdown measures limit the possibilities of injuries related to the movement of people and the pressure of patients with COVID-19 was never high during this period.

In orange wristbands, contradictory trends depend on the moment of the pandemic, with an increase in periods of more significant restriction (C1 and C2,1). During C1 the absence of established guidelines of treatment of the disease can explain this pattern as an infection could result in severe symptoms. The highest percentage of infected individuals is observed during C2,1 what explains a high number of orange wristbands [intensive care units reached in Portugal its highest level of occupation in the beginning of 2021 (11)]. This fact enhances the importance of taking measures before the number of infections start to grow exponentially. The percentage of orange wristbands is similar in C1 and C2,1 however the absolute number is quite different has access to ES was significantly higher during C2,1. On the other hand, non-urgent wristbands had a significantly higher percentage of occurrences in C2,1 and C2,2 than in the pre-pandemic period in all periods. The mean age of the infected individuals was lower in this period than it was during C1. Adding this to the process of vaccination which gave priority to older people (more susceptible to develop severe disease) and the wide acceptance of the vaccine originated a great number of non-severe cases what explains the high percentage of green wristbands among the COVID-19 positive individuals that can be observed in Figure 3 (11). Moreover, patients were often advised (albeit erroneously) to seek the ES for testing and confirmation of the diagnosis or for mild symptoms associated with COVID-19 infection that could be easily self-monitored at home. On the other hand, SNS24 telephone line was overload during this period which could make some people to go to an ES as it was not always SNS24 was not always available during this period. This line should be used before an individual with COVID-19 symptoms access to an ES (12).

In most of the studies available in the literature, there was a decrease in all priority codes, with the most accentuated reduction in the less urgent cases (2, 3, 5, 13–17). Portugal’s approach resulted in a different pattern, as the epidemic was very well contained when it emerged. In the second confinement, the combination of several factors (mean age of the infected individuals, vaccination process and overload of the SNS24 service) explains the high percentage of less urgent cases. Thus, this study obtained opposite results, which highlights the importance of analysing the results observed at CHUPorto as a case study that can provide lessons for a future pandemic. The results found are similar to the results found by Garcia (18) in a pediatric environment in Portuguese city of Coimbra.

Access to the ES was done, for the most part, on the individual’s own initiative. However, during the pandemic, there was a significant reduction in its percentage due to increased access through INEM and SNS24. During the 1st confinement, access originated SNS 24 service moved from 5th to 3rd position. This change in the pattern of access to the ES can be justified by the option of patients not resorting to the ES on their initiative and/or opting to contact the pre-emergency services such as the SNS24 telephone line. Moreover, calling SNS24 was often required to screen/confirm COVID-19 cases. Crossing the colour of the wristbands with the access variable allows us to corroborate this assumption as green episodes with origin in SNS24 increased (especially in periods C2,2 and P).

As for the demographic characteristics (gender and age) of ES users, the literature reports a decrease in admissions of young and female patients. Thus the user profile during the pandemic corresponds mostly to male and older patients (5, 13, 19–22). Our results corroborate these data with greater parity in demand for the two sexes (which contrasts with a higher prevalence of females in the pre-pandemic period) and an increase in the age average that occurs due to a decrease in demand by young adults.

The frequency of users going to an ES is associated with the ease and geographic proximity of the hospital to their residence (23, 24). The percentage of demand from Zone 1 has significantly increased. However, the percentage of demand from individuals living in Zone 2 (with a well-served transportation infrastructure and a reliable public transportation network) decreased, which indicates that factors related to the pandemic have induced some disturbance in what the usual demand from individuals residing in locations close to CHUPorto would be.

Regarding the outcome of emergency episodes (regular) discharges continued to be the most prevalent, although a significant decrease during the pandemic was verified. On the other hand, there is also a considerable increase in the percentage of supervised discharges and hospitalisations, which could be regarded as a better use of the ES as there is a need for follow-up/surveillance of the problem that motivated the visit to the ES. Discharges related to specialities of General Practice and Family Medicine are very rare during the pandemic, which may indicate lower demand for the resolution of less acute situations with consequences in the increase in the stock of disease. The COVID-19 patients’ internment was in a dedicated area of CHUPorto under the care of the Infectious Diseases service what explains the high increase in discharges related to this speciality. Another difference observed during the pandemic period was the percentage increase in the volume of deaths, especially in the most critical periods of the pandemic. This increase in mortality during the pandemic followed a national trend of rising death rates. The same increase in mortality in the ES has been reported in the literature (17, 25). This increase in hospitalisations and deaths may reinforce the idea that emergency episodes during the pandemic corresponded to more severe situations in terms of illness. Still, it could also be associated with the SARS-COV-2 disease, which, mainly before vaccination, caused a marked increase in intensive care and ward admissions and increased mortality associated with SARS-COV-2 infection.

The results regarding the evolution of behaviour in accessing the ES during the day and night should be read with caution. Zone 1 has some units with extended opening hours between 8 pm and 10:45 pm during the week and on weekends and holidays between 9 am and 6:45 pm. This effect was not considered in this analysis.

## Conclusion

The SARS-COV-2 pandemic has triggered challenges worldwide in various areas, from social, economic and health. Our study focused on analysing the impact of the pandemic on emergency services, which are at the forefront of any acute health issue. As noted in the literature, services are often problematic at the management level due to their dynamic characteristics and sensitivity to external factors. In the literature, the pandemic’s impact on access and the profile of ES users was evident. However, most studies refer only to the first year of the pandemic in which a new disease was treated.

Our results showed a significant reduction in admissions to the ES during the pandemic period compared to the same pre-pandemic period, with this reduction in hospitalisations being more pronounced during the first period of mandatory confinement. This reduction could explain the high mortality rates in Portugal that have remained since the beginning of the pandemic and why even the widespread vaccination of the population against the new coronavirus has not changed. Part of the explanation may lie in the increase in the stock of the disease in the Portuguese population, which can be observed (indirectly) by lower accesses to the ES, reduced accesses on their initiative or even by the specialities that attributed discharges during the pandemic period. Therefore, it is important, in future pandemics, to promote confidence in the use of health resources, even during periods of confinement. On the other hand, implementing measures before entering a phase of exponential growth in the epidemic curve is extremely important to avoid overloading the ES. Finally, it is advisable to have contingency plans for virtual assistance to individuals (telephone line, chatbot, …) to prevent people from overloading the ES in critical periods as it is a pandemic.

## Data availability statement

The data analyzed in this study is subject to the following licenses/restrictions: restrictions apply to the availability of these data, which were used under license for the current research and are not publicly available. Data are, however, available from the authors upon reasonable request and with permission of University of Porto Hospital Centre. Requests to access these datasets should be directed to jom@ess.ipp.pt.

## Ethics statement

The studies involving humans were approved by Comissão de Ética do CHUPorto|ICBAS, University of Porto Hospital Centre. The studies were conducted in accordance with the local legislation and institutional requirements. Written informed consent for participation was not required from the participants or the participants' legal guardians/next of kin in accordance with the national legislation and institutional requirements. Written informed consent was not obtained from the individual(s) for the publication of any potentially identifiable images or data included in this article because The research involves the use of existing databases.

## Author contributions

AN: Writing – original draft. CC: Writing – original draft. JM: Writing – original draft. PF: Writing – review & editing. RP: Writing – review & editing.
